# Light-microgel interaction in resonant nanostructures

**DOI:** 10.1038/s41598-018-27197-4

**Published:** 2018-06-19

**Authors:** M. Giaquinto, A. Ricciardi, A. Aliberti, A. Micco, E. Bobeico, M. Ruvo, A. Cusano

**Affiliations:** 1 Optoelectronics Group, Department of Engineering, University of Sannio, I-82100 Benevento, Italy; 2 ENEA, Portici Research Center, P.le E. Fermi 1, I-80055 Portici, Napoli, Italy; 3 Institute of Biostructure and Bioimaging, National Research Council, I-80143 Napoli, Italy

## Abstract

Combination of responsive microgels and photonic resonant nanostructures represents an intriguing technological tool for realizing tunable and reconfigurable platforms, especially useful for biochemical sensing applications. Interaction of light with microgel particles during their swelling/shrinking dynamics is not trivial because of the inverse relationships between their size and refractive index. In this work, we propose a reliable analytical model describing the optical properties of closed-packed assembly of surface-attached microgels, as a function of the external stimulus applied. The relationships between the refractive index and thickness of the equivalent microgel slab are derived from experimental observations based on conventional morphological analysis. The model is first validated in the case of temperature responsive microgels integrated on a plasmonic lab-on-fiber optrode, and also implemented in the same case study for an optical responsivity optimization problem. Overall, our model can be extended to other photonic platforms and different kind of microgels, independently from the nature of the stimulus inducing their swelling.

## Introduction

Microgels (MGs) are colloidal hydrogel microsized particles with radius in the range between tens of nanometers and a few microns^1–8^. In the event of chemical or physical stimuli due to temperature, pH, ionic strength variations, or molecular binding, MGs immersed in a liquid environment respond with a change of their size, individual chain dimensions, solubility or the degree of intermolecular association^9^. Thanks to these unique properties, MGs are effectively used for sensing purposes^10^, since they can be synthesized in order to be responsive to a specific parameter of interest. In biological sensing field, functionalized MGs offer the possibility to increase the target analyte loading capacity (by translating a 2D interaction surface into a 3D volume), and amplify the optical signal transduction^11–14^.

Typically, MGs dissolved in liquid solutions are interrogated with bulky readout systems (optical microscopy, confocal fluorescence microscopy, dynamic light scattering analysis) able to detect and quantify the size variations as a function of external stimulus applied. Recently, with the development of suited deposition strategies^15–18^, MGs have been integrated with resonant *optical* devices, where the resonance wavelengths are modulated by the MGs properties changes^19–23^. Concerning planar chip, a Fabry-Perot etalon has been demonstrated by sandwiching a MGs film between two thin gold layers. Variations in the optical length of the cavity cause a change in the phase of the interference wave arising from light reflected at the substrate-gel and gel-solution interfaces^19,20^. On the other hand, MGs films have been also integrated on Lab-on-Fiber (LOF) based probes, opening the way also for *in-vivo* applications, thanks to integration of optical fibers with medical needles or catheters^24^. Specifically, MGs have been deposited over a metallic nanostructure backed by the tip of a standard single mode optical fiber^21–23^. In this case, the plasmonic resonance wavelength shifts as a function of the MGs film swelling/shrinking induced by temperature and molecular binding.

The intrinsic nature of MGs makes their interaction with resonant modes not obvious; in fact, it is known that MGs refractive index (RI) increases/decreases when their size decreases/increases^25^. As a consequence, the enormous potentialities arising from the MGs integration onto the resonant nanostructures can be exploited only through the implementation of suitable models able to describe the relationship between geometrical (size) and physical (RI) characteristics of the MGs layer deposited on a surface, as a function of the specific stimulus applied.

So far, thickness and RI of surface-attached MGs have been measured *separately*, by exploiting different approaches. On one hand, thicknesses of MGs films are measured by means of optical waveguide spectroscopy^26^, z-scans in confocal fluorescence microscopy^17^ and atomic force microscopy (AFM) in liquid solution^27^. Particle swelling kinetics can be also estimated with chemo-electro-mechanical theoretical models, based on coupled nonlinear partial differential equations requiring moving boundary conditions^28^. On the other hand, MGs RI can be determined by combining measurements of the volume fraction of the MGs suspension, with RIs of both the suspension and the surrounding medium^29,30^. Specifically, the volume fraction can be derived experimentally from dynamic light scattering (DLS) and viscosimetry measurements^31^, while the RIs can be measured by means of an Abbe refractometer. It becomes clear that the entire procedure is quite tricky and require high efforts in terms of time and costs, since it needs to be repeated for different values of temperatures and MGs concentrations. Moreover, although the above mentioned techniques allow to determine the *single* MG characteristics, they do not provide information about the MGs *film* optical properties. In fact, the MGs layer degree of compactness of particles over the surface strongly influence the final device performances^22^.

**Figure 1:**
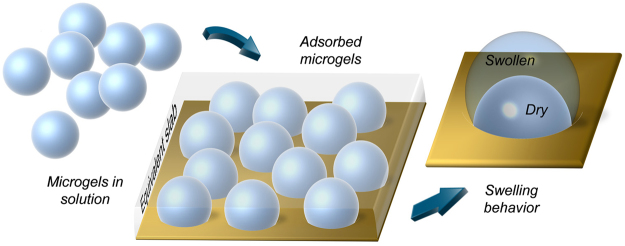
Microgels adsorbed on a substrate are modeled as an equivalent dielectric slab whose thickness and RI are a function of the swelling state.

In this framework, here we propose an analytical approach for modeling the optical properties of MGs film deposited on a resonant surface. MGs layer is considered as an equivalent dielectric slab, (Fig. 1) whose characteristics also depend on the degree of compactness and the distribution of MGs. The slab RI and thickness are thus related to the MGs properties directly derivable from *conventional* morphological analysis, such as DLS (in liquid) and AFM (in air) measurements. Without loss of generality, the proposed model is validated by comparing numerical and experimental results pertaining to LOF probes integrated with MGs layers with different degree of compactness and uniformity. In our validation experiments, we have used temperature as a benchmark stimulus, because it is an absolute value, which can be univocally determined. However, our model is valid independently from the stimulus applied, since it is based on experimental observations (i.e. DLS and AFM measurements) which intrinsically take into account the effect of the stimulus on the MG particle. In this regard, we have found that temperature variations or molecular binding events induce the same MG volume phase changes at the steady state^21^.

The rest of the paper is divided in four main sections. The first one is an experimental part dealing with fabrication, morphological and optical characterization of the MGs assisted LOF probes (details on the experimental procedures are included in the methods, at the end of the manuscript). In section II we introduce the MGs slab model, while in section III we demonstrate its validity in the analyzed case study. Finally, in section IV, we implement the model in an optical responsivity optimization problem.

## Results and Discussion

### MGs assisted LOF probe: fabrication and characterization

LOF technology is generally employed to realize onto flat tip of optical fibers metallic or dielectric nanostructures supporting resonant modes that are sensitive to local RI changes of the environment surrounding the fiber^32–36^. In this study, LOF technology is exploited for integrating on the tip of a standard single mode optical fiber, a 35 nm thick gold layer patterned with a square lattice of holes with period and radius equal to 680 nm and 190 nm respectively. The realized nanostructure supports at specific wavelengths plasmonic modes arising from the coupling of localized surface plasmonic resonances (LSPR), due to the presence of subwavelength holes, and surface plasmon polaritons (SPP), typically excited on continuous metallic layers. Specifically, the incident light couples to the SPP through the grating coupling, that propagates along the metallic layer and is scattered by the holes, enabling the so called “extraordinary optical transmission” (EOT)^37^. In the wake of our previous work^22^ we chose the pattern period for setting the resonant wavelength is in the operating range of standard single mode fibers; a filling factor (radius to pitch ratio) of 0.3 allows to maximize the absolute wavelength shift induced by the change of the surroundings, at the expense of a narrowband spectral feature.

**Figure 2:**
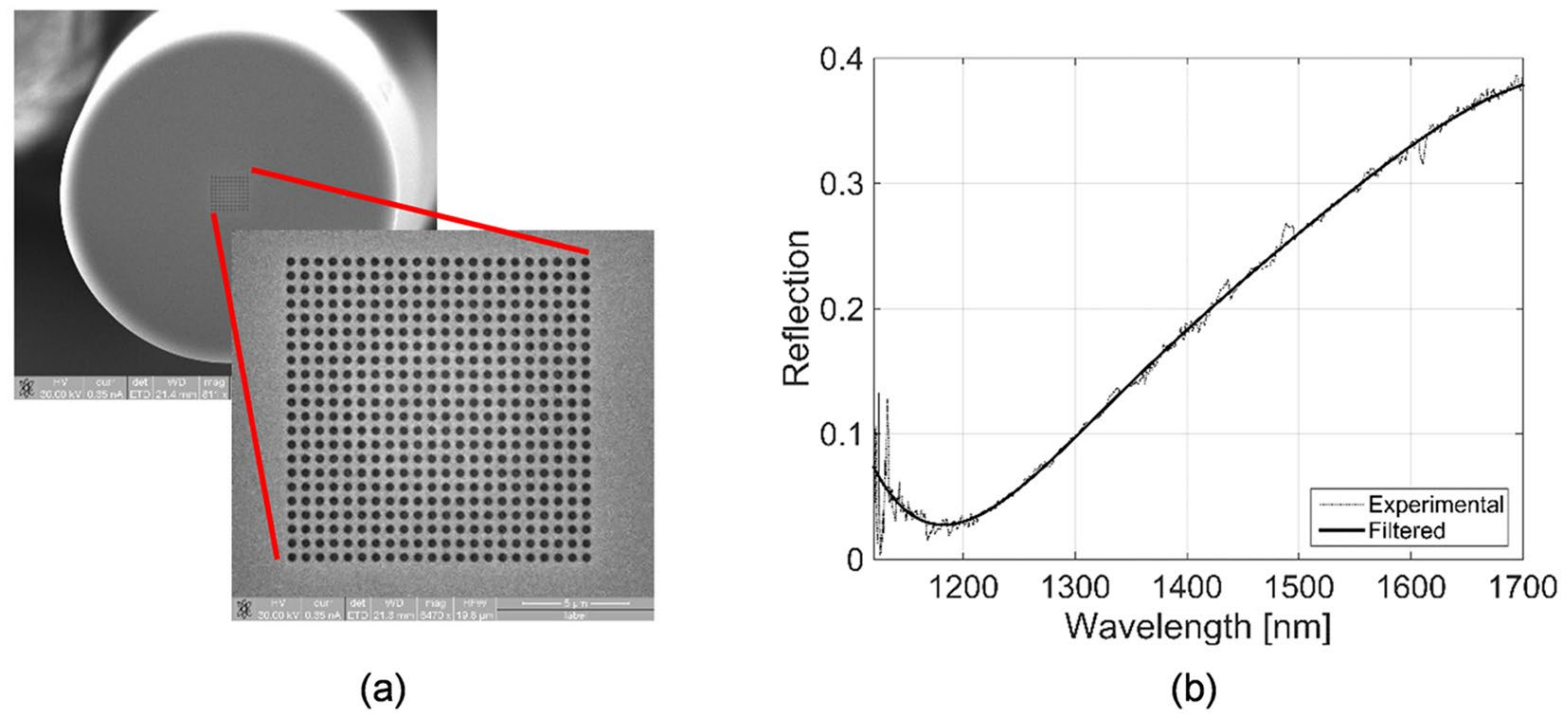
SEM image (top view) (**a**), and reflection spectrum in air (**b**) of the LOF probe before MGs deposition.

The fiber probe has been realized by directly milling the gold layer via a focused ion beam (FIB) process^38^. Details on the fabrication procedure are included in the methods. Figure 2a shows a top view SEM image of the fiber facet. The probe was optically characterized in the optrode configuration. Experimental reflection spectrum (Fig. 2b) shows a dip at around 1180 nm due to the excitation of a surface plasmon resonance^37^.

**Figure 3:**
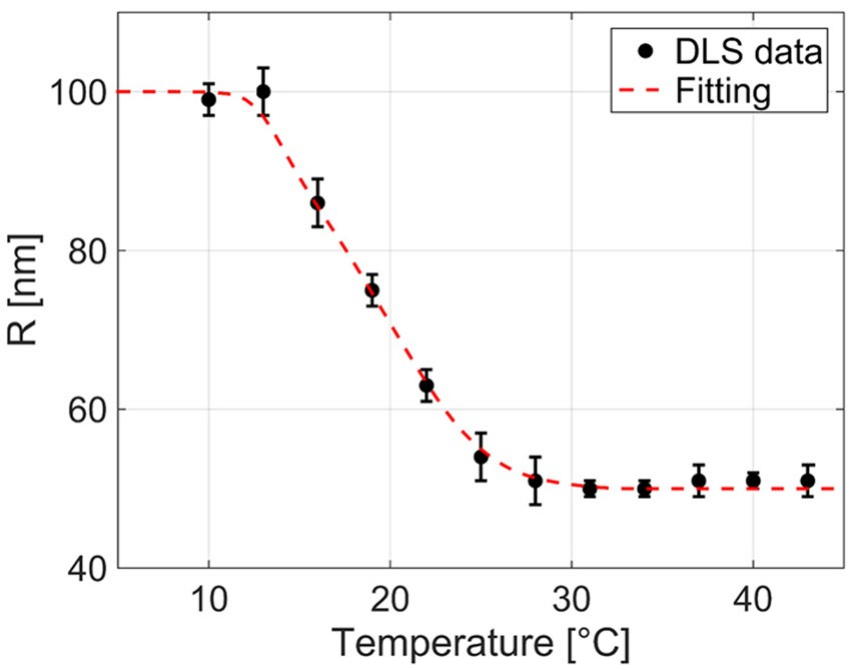
Hydrodynamic radius (R) of the (pNIPAm-APBA) MGs as a function of temperature (black points) fitted with a smoothing spline (red dashed curve). Error bars represent one standard deviation.

pNIPAM-based responsive MGs were synthesized following an approach described in ref.^19^. MGs were successively characterized by means of DLS measurements in a buffer solution (pH 9), in order to derive the relationship between the hydrodynamic radius *R* and temperature *T*. Details on DLS measurements are included in the methods. The DLS measurements results are shown in Fig. 3; experimental data are interpolated by a smoothing spline (red dashed curve). The total radius variation is about 50 nm in the temperature range 10–43 °C. For T > 13 °C, *R* linearly decreases with a slope of about 3.7 nm/°C, before saturating at ~26 °C.

**Figure 4:**
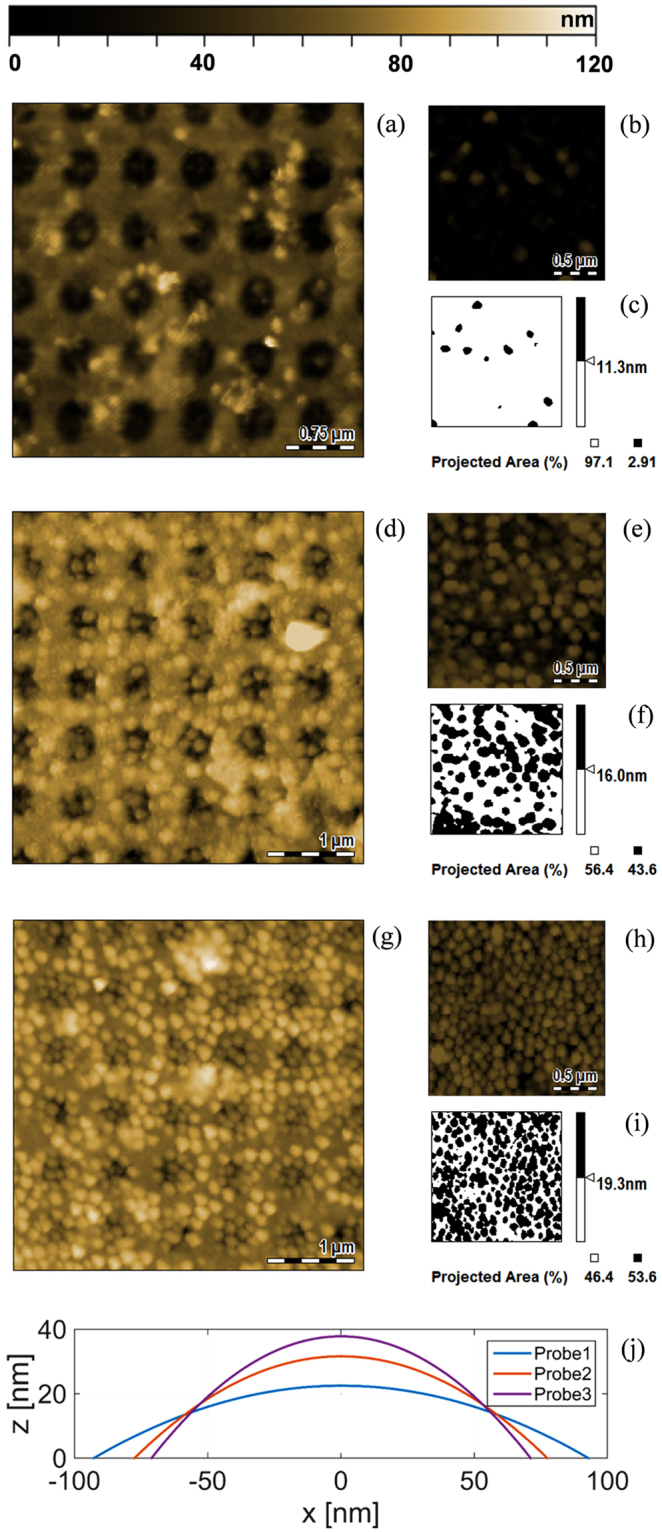
AFM analysis of the optical fiber core region (4 µm × 4 µm top view) of the probes fabricated with MGs concentrations of 0.01% (**a**), 0.05% (**d**) and 0.5% (**g**). AFM images of unpatterned region (2 µm × 2 µm area) of the LOF probes fabricated with MGs concentrations of 0.01% (**b**), 0.05% (**e**) and 0.5% (**h**,**c**,**f**,**i**) slice processing of the MGs thickness of figures (**b**,**e**) and (**h**). Single MG profiles averaged over 10 profiles measured on different areas, and fitted with a quadratic curve (**j**).

MGs were deposited onto the fiber tip by means of a dipping procedure consisting in immersing the fiber in a buffer solution containing a specific concentration of MGs (see Methods for more details). In our study, we have used three different MGs concentrations in solution for realizing three different probes, namely Probe 1, Probe 2 and Probe 3. Specifically Probe 1 was realized with a MGs concentration of 0.01%, Probe 2 with a MGs concentration of 0.05% and Probe 3 with a MGs concentration of 0.5%. This is because MGs concentration strongly affects the compactness and density of the deposited layer. With reference to Fig. 4a,d,g, in agreement with our previous work^22^, we found that for concentration of 0.5%, MG particles form a compact layer which is conformally deposited onto the patterned structure. By decreasing the concentration, the particles become gradually sparser on the surface. This effect can be quantified by means of a threshold analysis (evaluated at the MGs half-height) of AFM images pertaining to MGs covering unpatterned areas (Fig. 4b,e,h). Figure 4c,f,i reveal a MGs surface occupation of 3%, 44%, and 54% for MGs solutions of 0.01%, 0.05%, and 0.5% respectively. Moreover, for each probe, the single MG profile has been retrieved from a mean over 10 profiles measured over different areas. The average profiles are shown in Fig. 4j, fitted with a quadratic curve.

It is interesting to observe that, by increasing the MGs concentration in solution, the deposited MGs thickness increases with a consequent reduction of the base radius. In fact, by increasing MGs concentration, the repulsion forces among MGs deposited on the gold surface prevail on MGs-Au interactions, thus determining an increase of MGs thickness film^27^. Fig. 4 shows that MG particles are deposited also inside the nanostructure holes; this is something useful for maximizing the device responsivity since the areas inside the holes are characterized by the strongest field localization^39^.

**Figure 5:**
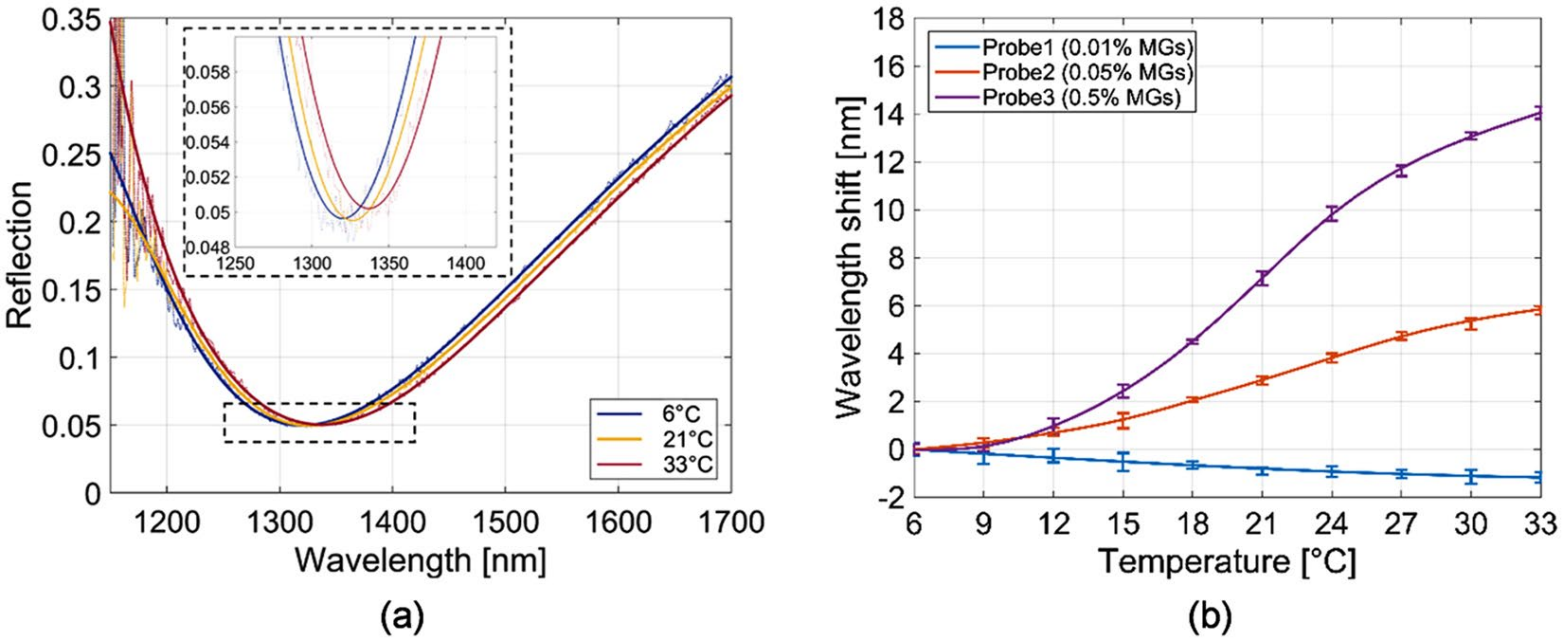
(**a**) Experimental reflection spectra pertaining to the probe fabricated with a concentration of MGs of 0.5% at temperatures of 6, 21 and 33 °C. (**b**) Resonance wavelength shift vs temperature of the LOF probes integrated with MGs at different concentrations: 0.01% (Probe1), 0.05% (Probe2), 0.5% (Probe3). Error bars represent one standard deviation. Solid curves are obtained through a smoothing spline fitting method.

After MGs integration, the probes have been optically characterized for determining the effect induced by temperature on the resonant wavelengths. To this aim, the probes were immersed in a cuvette containing a pH 9 buffer solution, whose temperature was controlled by means of a Peltier Cells based system^22^. The temperature was changed in a range between 6 °C and 33 °C with a step of 3 °C. Five reflection spectra were recorded (1 per minute) for each temperature. As representative case, Fig. 5(a) shows the reflection spectra pertaining to the probe fabricated with a concentration of MGs of 0.5% measured at 6, 21 and 33 °C. Figure 5(b) shows the measured resonant wavelength shifts (pertaining to the reflection spectra minima) as a function of temperature for the 3 probes.

According to the plasmonics theory, the resonance shifts to longer wavelength (lower energy) when the RI or the thickness of the environment is increased^40,41^. Interestingly, here we found that the wavelength shift driven by the MGs swelling state has amplitude and sign that depends on MGs film properties. In fact, the probe fabricated starting from a MGs concentration of 0.01% responds to the temperature increase with a wavelength *blue-shift* of about 1.5 nm. The validity of these findings have been confirmed through repeatability tests. The remaining probes respond to the temperature increase with a wavelength *red-shift*, whose amplitude increases according to MGs concentration. According to DLS measurements (Fig. 3), the temperature increase causes a shrinking of the MGs layer (thickness reduction) with the consequent increasing of its equivalent RI. As a consequence, the large resonance red-shift, demonstrates that the effect due to RI prevails on that due to thickness, as a thickness reduction would have caused a resonance blue-shift^41^. In the case of Probe 1, the MG layer does not influence the probe response in a significant manner since the particle distribution on the fiber tip is rather sparse; the resonance blue shift could be thus caused by the thermo-optic effect of both the NIPAM and the buffer solutions in which the probes are immersed; in fact, both polymers and liquids have negative thermo-optic coefficient^42,43^. With the aim of investigating the dependence of the resonant wavelength on the MGs layer properties, in the following, we describe a simple model, where the MGs film is considered as a uniform dielectric layer conformally deposited on the metallic grating. The MGs slab equivalent thickness *h*_*slab*_ and RI *n*_*slab*_ are a function of MGs swelling/shrinking states.

### Modelling MGs slab

The hereinafter discussed model has been developed with the aim of expressing the MGs slab properties as a function of a few key geometrical parameters that could be directly referable to conventional morphological analysis. To make the discussion streamlined, details on the mathematics are included in the supporting information.

#### MGs slab refractive index

The volume of swollen MG in solution *V* is given by the sum of volumes of the constituting materials, i.e. the volumes of the polymer *V*_*p*_ and that of the fully incorporated liquid solution *V*_*s*_, i.e.

1 $${V}={{V}}_{{p}}+{{V}}_{{s}}$$

Since the elements constituting MGs have dimensions much smaller than the wavelengths, MGs RI *n*_*MGs*_ can be expressed as a weighted average of those of polymer *n*_*p*_ and buffer solution *n*_*s*_:

2 $${n}_{MGs}=\frac{{n}_{p}{V}_{p}+{n}_{s}{V}_{s}}{V}$$

**Figure 6:**
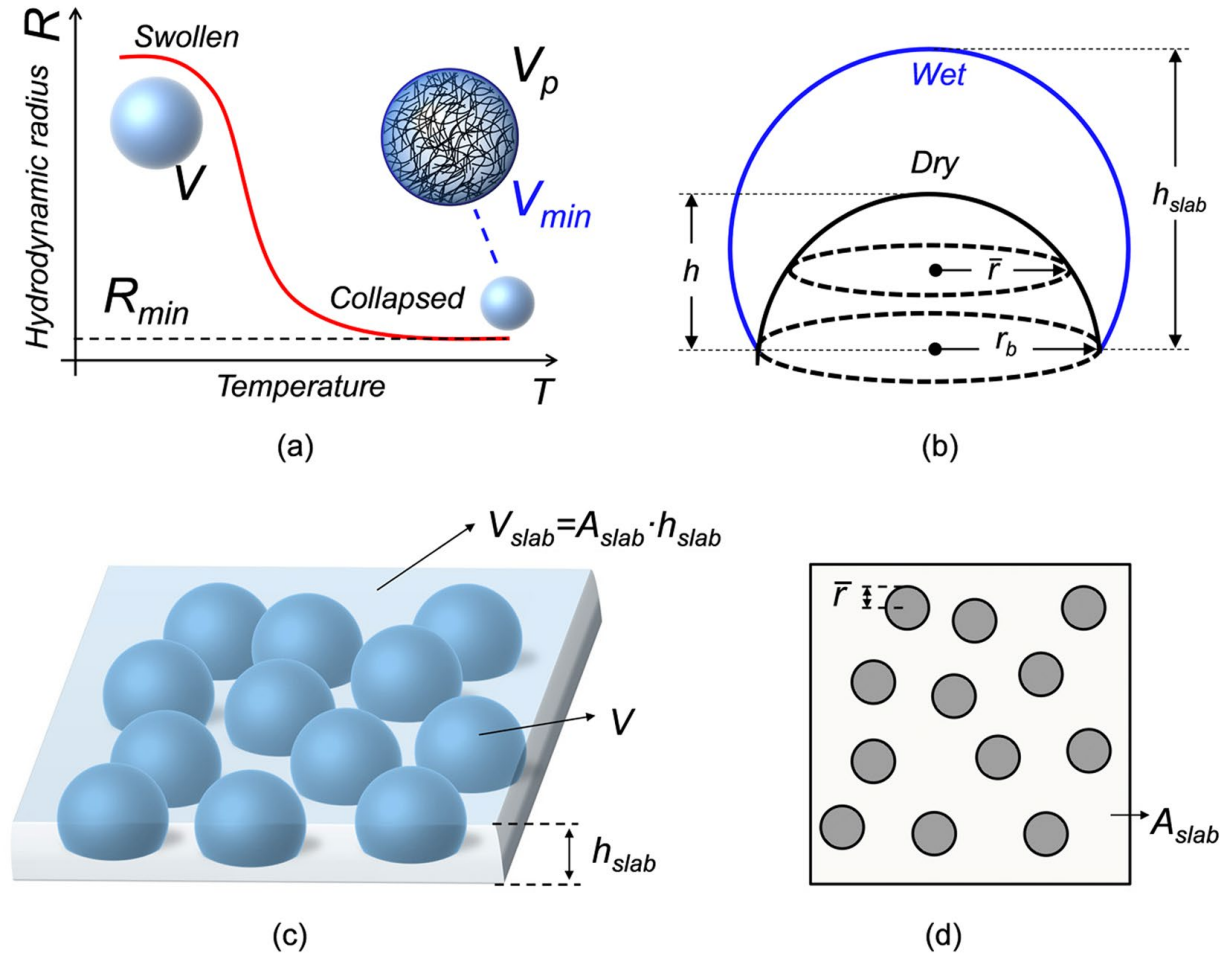
(**a**) Schematic of MGs swelling in solution where particles have a spherical shape with radius R and volume V. V_p_ is the polymer volume and V_min_ is the MG particle volume in the collapsed state. (**b**) The profile of the surface-attached MG particle is modeled as a spherical cup with base radius r_b_ (in both the wet and dry state), height h (in the dry state) and h_slab_ (in the wet state). (**c**) Schematic of the slab formed by surface attached MGs on the surface in the wet state: V is the single particle volume (i.e. spherical cup) while V_slab_ indicates the box volume. Note that V is the same quantity in images (**a**,**c**). (**d**) Cross-section of (**c**) at half-height in the dry state; black circles with radius $\bar{r}$ indicate the single MG particle boundaries.

Eq. (2) is slightly manipulated in order to express *n*_*MGS*_ as a function of parameters derivable from conventional morphological analysis. With this aim, by defining *V*_*min*_ as the volume of (wet) MGs in the collapsed state, then *V*_*p*_ is expressed as:

3 $${V}_{p}=\alpha {V}_{{\rm{\min }}}$$

where *α* (defined in the range 0–1) is the *polymer fraction* parameter (Fig. 6a); basically, when *α* = 1 (unrealistic case) the MGs in the collapsed state are only composed by polymer (*V*_*s*_ = 0). Consequently, by combining eqs (1) and (3) we achieve:

4 $${V}_{s}=V-\alpha {V}_{{\rm{\min }}}$$

By substituting Eqs (3) and (4) in (2), and by assuming that MGs suspended in liquid solution have a spherical shape (Fig. 6a), we obtain that (see S1 in supporting information):

5 $${n}_{MGs}(R)=\alpha \cdot ({n}_{p}-{n}_{s})\cdot \frac{{R}_{{\rm{\min }}}^{3}}{{R}^{3}}+{n}_{s}$$

where *R* and *R*_*min*_ are the MGs hydrodynamic radii in the swollen and collapsed state respectively. These two radii values can be directly evaluated by DLS measurements.

A more detailed discussion is necessary for defining the RI (*n*_*slab*_) of the MGs *slab* formed by a closed-packed assembly of particles attached on a substrate. Clearly, *n*_*slab*_ strongly depends on the distribution and compactness degree of particles. For taking into account this effect, we introduce a *coverage factor γ* (defined in the range 0–1) describing the MGs volumetric density on the substrate. By assuming that, for each swelling state, the total volume and thus the RI of the single MG remains unaltered after deposition, *n*_*slab*_ is thus expressed as:

6 $${n}_{slab}=\gamma \cdot {n}_{MGs}+(1-\gamma )\cdot {n}_{s}$$

By substituting (5) in (6), the above equation becomes (see S2 in supporting information):

7 $${n}_{slab}(R)=\gamma (R)\cdot \alpha \cdot ({n}_{p}-{n}_{s})\cdot \frac{{R}_{{\rm{\min }}}^{3}}{{R}^{3}}+{n}_{s}$$

A comparison between Eqs (5 and 7) indicates that *n*_*slab*_(*R*) has the same expression of *n*_*MGs*_(*R*) except for *α* that is scaled by the factor *γ*.

#### MGs slab thickness

With reference to Fig. 6b,c, *h*_*slab*_ corresponds to the height of the single MG deposited on the substrate. Such a parameter is related to the MGs hydrodynamic radius *R* through a *thickness growth parameter β*, whose value changes as function of the MGs swelling state, *i.e*.:

8 $${h}_{slab}=\beta (R)\cdot R$$

When *β* tends to 0, so does *h*_*slab*_, and MGs tend to be completely crushed on the substrate. On the other hand, when *β* = 1, *h*_*slab*_ = *R*. When MGs assume the original spherical shape*, β* tends to a value close to 2. It is important to remark that, when MGs are very packed among them, as experimentally observed with AFM characterization (Fig. 4), β can also assume values higher than 2.

Eqs (7) and (8) allow to express *n*_*slab*_ and *h*_*slab*_ as a function of the hydrodynamic radius of the single MG in solution *R*. These relationships include *α*, *β* and *γ* that can be estimated through a morphological analysis. As a matter of fact, *α* can be rigorously determined by capillary viscometry measurements in the diluted regime at different MGs concentrations and temperatures^31,44^. The parameters *β* and γ can be estimated through AFM measurements in a liquid environment. However, this kind of measurements is not easy to perform due to the presence of liquid; moreover, *β* and *γ* are also function of the external stimuli inducing MGs swelling process. This implies that AFM measurements should be repeated at different swelling states, making the characterization more consuming in terms of time and costs. Based on these considerations, in the following paragraph, we propose a method for estimating *α*, *β* and *γ*, starting from DLS (in buffer solution) and AFM measurements (in air), i.e. when the MGs are in the dry state on the substrate.

#### Relationship between MGs slab properties and morphological analysis data

By keeping the assumption that the total volume of a single MG is retained after the deposition, whatever its swelling state, and by considering that the polymeric volume *V*_*p*_ cannot change after the deposition (*i.e*. the single deposited MG in dry state has a volume *V*_*p*_) the parameter *α* can be simply derived from Eq. (3). *V*_min_ is directly calculated from DLS analysis, by considering the MG particle in liquid as a sphere of radius *R*_min_ (i.e. the hydrodynamic radius in the collapsed state). *V*_*p*_ is evaluated by assuming that the surface-attached MG in air has the form of a spherical cap, with base radius *r*_*b*_ and height *h*. Note that both *r*_*b*_ and *h* can be directly estimated by AFM analysis of deposited MGs in air (see Fig. 4j). By substituting expressions of volumes *V*_*p*_ and *V*_min_ in Eq. (3), we obtain (see S3 in supporting information for details):

9 $$\alpha =\frac{{h}^{3}+3h{r}_{b}^{2}}{8{R}_{{\rm{\min }}}^{3}}$$

The coverage factor *γ*(*R*) is defined as the ratio between the total MGs *volume* in a slab (i.e. the single MG volume multiplied by the number (*N*_*MG*_) of MGs attached over an area *A*_*slab*_), and the overall volume of the equivalent slab defined as *V*_*slab*_ = *A*_*slab*_ *h*_*slab*_ (see Fig. 6c). *N*_*MG*_ and *A*_*slab*_ can be expressed as a function of the radius $\bar{r}$ and the *superficial* coverage factor $\bar{\gamma }$ of surface-attached MGs evaluated at half height, directly measured by means of AFM analysis (see Figs 4c,f,i and 6d). This finally leads to the following relation (see S4 of supporting information for details):

10 $$\gamma (R)=\frac{4}{3}{(\frac{R}{\bar{r}})}^{2}\frac{\bar{\gamma }}{\beta (R)}$$

As expected, *γ* is a function of *β*. In the particular case of *γ* = 1, *β* can be directly deduced from Eq. (10), independently from the shape assumed by the single deposited MGs:

11 $$\beta (R)=\bar{\gamma }\frac{4}{3}{(\frac{R}{\bar{r}})}^{2}$$

Moreover, for evaluating *β* when *γ* < 1, each MG particle is modeled as a spherical cap, whose volume corresponds to that measured at DLS (for each swelling state). Since the base radius changes are negligible with respect to the height variations during the swelling process^27,45^, the spherical cup base radius is kept constant to *r*_*b*_. In the wake of these considerations, *h*_*slab*_ (and consequently *β* = *h*_*slab*_*/R*) in the swollen state corresponds to the solution of the following equation (see S5 in supporting information):

12 $${h}_{slab}^{3}+3{r}_{b}^{2}{h}_{slab}-8{R}^{3}=0$$

eq. (12) admits only one real solution, which is positive when both *R* and *r*_*b*_ are positive:

13 $${h}_{slab}={(4{R}^{3}+\sqrt{{r}_{b}^{6}+16{R}^{6}})}^{1/3}-\frac{{r}_{b}^{2}}{{(4{R}^{3}+\sqrt{{r}_{b}^{6}+16{R}^{6}})}^{1/3}}$$

It is interesting to observe that, when *R* approaches *r*_*b*_, eq. (13) becomes approximately linear; as a consequence, it can be simplified by considering the first order of Taylor series evaluated around *r*_*b*_(see S5 in supporting information). The final expression of *β* = *h*_*slab*_/*R* thus becomes:

14 $$\beta (R)\approx \{\begin{array}{cc}{\rm{2}}.\,{\rm{4}}-0.92\,\frac{{r}_{b}}{R}, & \gamma  < 1\\ \bar{\gamma }\frac{4}{3}{(\frac{R}{\bar{r}})}^{2}, & \gamma =1\end{array}$$

According to Eqs (8) and (14), while *γ* < 1, *h*_*slab*_ increases almost linearly with *R*, with a slope of about 2.4. The value of *r*_*b*_ affects the linear trend offset. Note that, coherently with what has been mentioned before, *β* approaches 2 when *r*_*b*_ << *R*, i.e. when the deposited MG shape tends to be spherical (the slight overestimation is due to the linear approximation). On the other hand, when *γ* = 1, i.e. when MGs particles are very packed among them to form a compact slab, *h*_*slab*_ increases with the cube of *R*.

Overall, in Eqs (9), (10) and (14) the parameters *α, β* and *γ* are expressed as a function of quantities that are directly derivable from DLS (in the liquid state) and AFM (in the dry state) measurements. The combination of (7) and (8) with (9), (10) and (14) represents a complete set of equations for modelling the optical properties of the MGs slab deposited on a surface. In the next section, we validate the developed model by applying it to the experimental case study previously discussed.

### Validation of MGs slab model: comparison between experimental and numerical data

From previous morphological analysis, for each probe, it is possible to derive *h*, *r*_*b*_, $\bar{r}$, and $\bar{\gamma }$, and consequently, calculate the parameters *α*, *β* and γ. Specifically, the geometrical parameters *h, r*_*b*_ *and* $\bar{r}$ are directly derived from the surface-attached MGs particles profiles shown in Fig. 4j, while the parameter $\bar{\gamma }$ is derived from the AFM data slice processing shown in Fig. 4c,f,i. The results are resumed in Table 1 where, being *β* and γ variables, we included just their minimum and maximum values.

**Table 1:** AFM morphological parameters of Probe1, Probe2 and Probe3, and correspondent values of α, β and γ evaluated with the proposed model.

Probe		AFM morphological parameters				Evaluated parameters				
	MGs Conc.	*h* [*nm*]	*r*_*b*_ [*nm*]	$\bar{r}$ [*nm*]	$\bar{\gamma }$ [*a.u*.]	*α* [*a.u*.]	*β*_*max*_ [*a.u*.]	*β*_*min*_ [*a.u*.]	*γ*_*max*_ [*a.u*.]	*γ*_*min*_ [*a.u*.]
Probe1	0.01%	22.50	92.75	65.38	0.03	0.59	1.58	0.74	0.05	0.03
Probe2	0.05%	31.60	77.42	54.66	0.44	0.60	1.92	0.98	1	0.49
Probe3	0.5%	37.03	71.12	50.15	0.54	0.61	3.24	1.10	1	0.73

For the function β and γ only the minimum and maximum values are shown.

From Table 1, several considerations emerge; as expected, since the probes are realized starting from the same kind of MGs, *α* is approximately the same in all the 3 cases (~0.6). On the other hand, *β* and *γ* strongly depend on the MGs degree of compactness on substrate. In fact, for each probe, when MGs are collapsed at high temperatures, they do not interact with each other (*γ* < 1) and *β* assumes values close to 1^16^. With reference to Probe1, *β* reaches values lower than 1 due to the strong MGs-Au interaction given by the sparse distribution of particles on substrate. On the other hand, in Probe3, *β* has values higher than 2, because of the MGs-MGs interaction due to high compactness of particles on the substrate.

**Figure 7:**
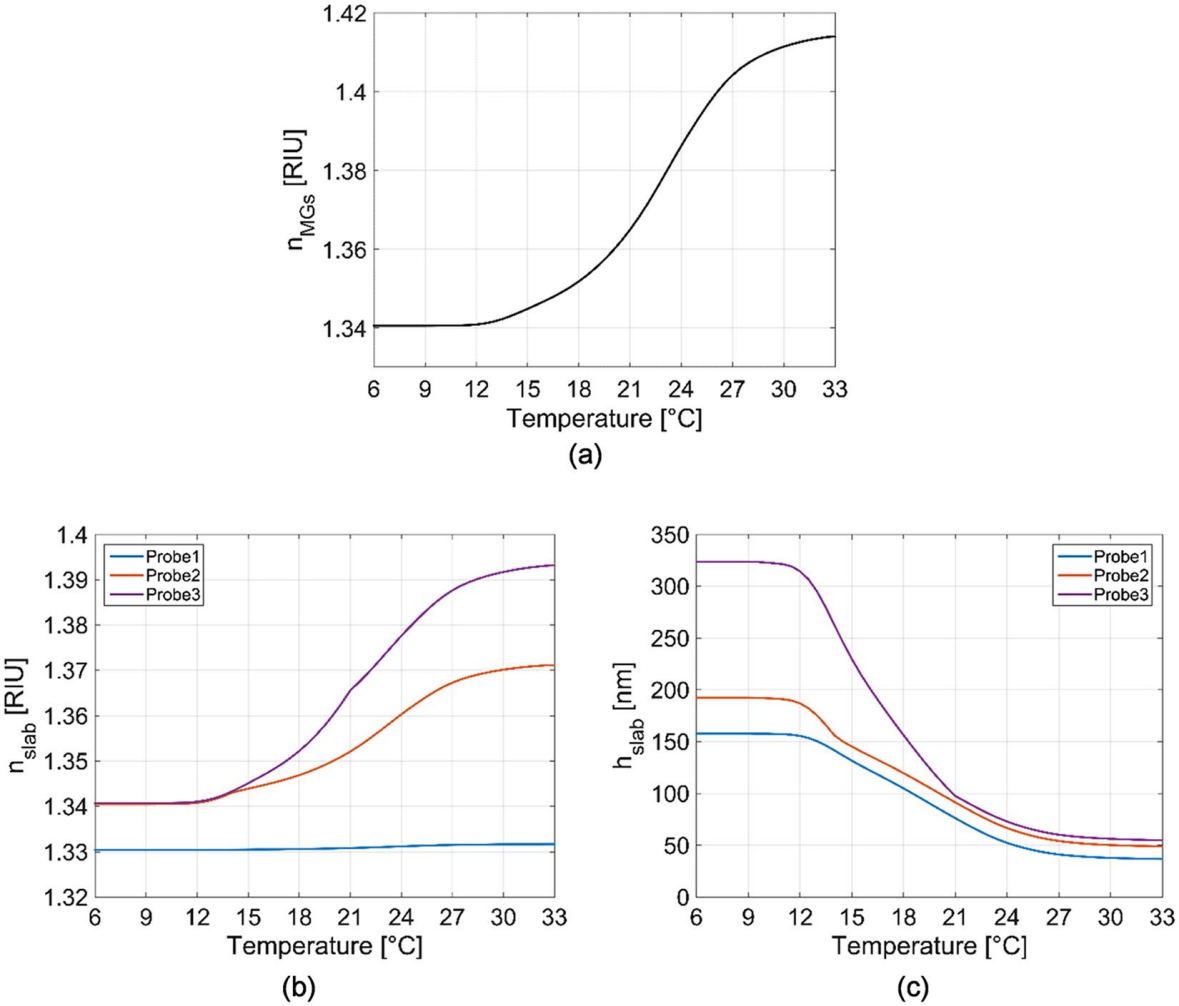
Single MG particle RI as function of temperature (**a**), MGs slab RI (**b**) and thickness (**c**) as function of temperature, theoretically evaluated according to the proposed model.

First, from eq. (5), it is possible to calculate the single MG RI (*n*_*MGs*_) in solution as a function of its hydrodynamic radius *R(T)* at different temperatures. *n*_*p*_ and *n*_*s*_ were set to 1.33 (water) and 1.47 (pNIPAM)^46^ respectively. Influence of both the cross-linker and the functionalizing molecules on *n*_*p*_ is neglected^27^. *R*_*min*_ was set to 50 nm, that is the minimum of the function *R(T)* shown in Fig. 3, while *α* was 0.6. As shown in Fig. 7a, *n*_*MGs*_ exhibits an increasing trend over the temperature, assuming values ranging from 1.34 to 1.414, with maximum slope of about 0.0075 RIU/°C at ~23 °C. It is interesting to note that *n*_*MGs*_ curve is both qualitatively and quantitatively coherent with experimental data achieved through ellipsometry measurements described in other works^47,48^. Successively, from eqs (7) and (8), we evaluate *n*_*slab*_ and *h*_*slab*_ as function of the temperature (Fig. 7b,c). Figure 7b confirms that *n*_*slab*_ pertaining to Probe1 has a constant value of about 1.33 (buffer solution RI) due to the fact that *γ* ≈ 0. RI curves of Probe2 and Probe3 are superimposed at lower temperatures (as *γ*_*max*_ = 1 in both the cases) and they gradually depart from each other as the temperature is increased. Differences among the 3 curves in Fig. 7c are more evident at lower temperatures (larger differences of *β*_*max*_) because of the MGs-MGs interaction effects.

**Figure 8:**
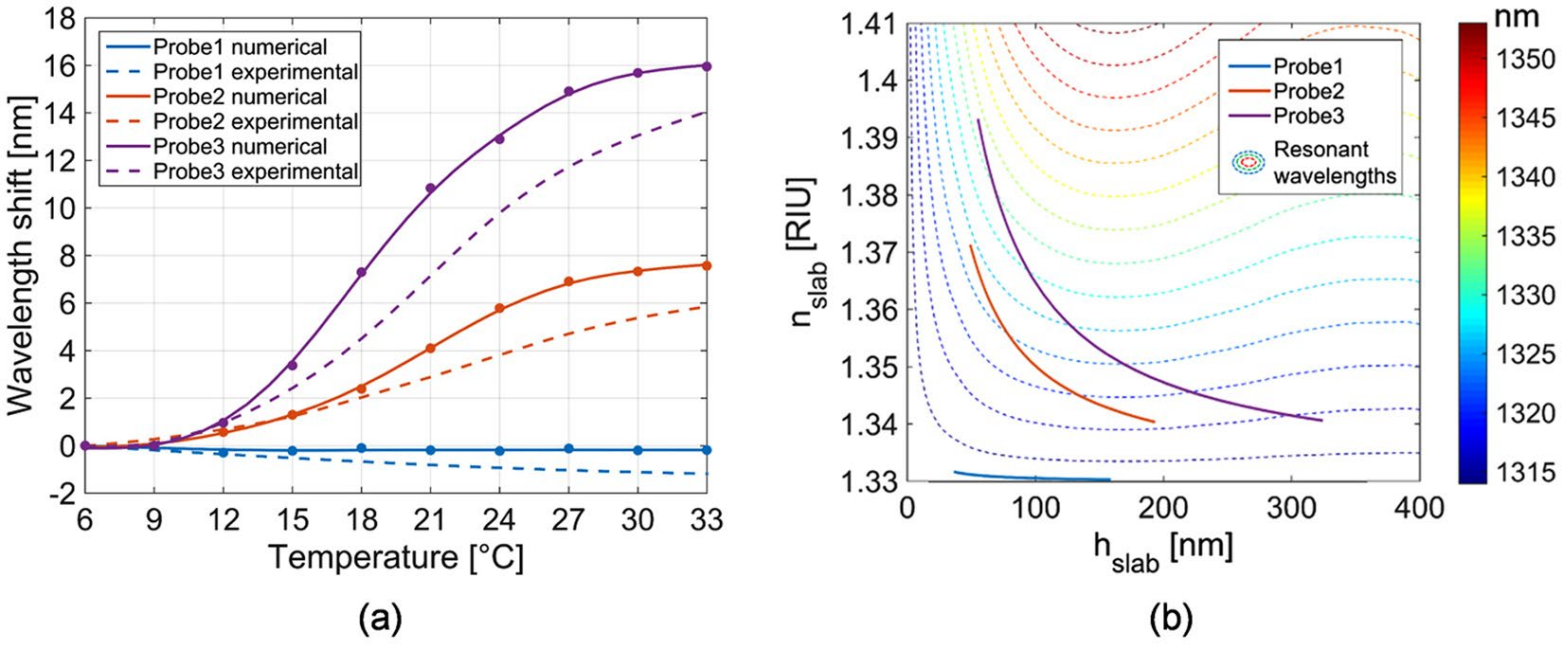
(**a**) Numerical wavelength shift as function of temperature numerically evaluated by using n_slab_ and h_slab_ reported in Fig. 7, and compared with the experimental results (dashed curves) already shown in Fig. 5b. Numerical results (dots) were fitted with smoothing spline method (solid curves). (**b**) Functions n_slab_ (h_slab_) evaluated for the three analyzed probes by using the proposed model. The three pathlines are superimposed to a contour plot (dot lines) showing the numerically evaluated resonant wavelengths as function of dielectric slab thickness h_slab_ and RI n_slab_.

Once known the functions *n*_*slab*_*(T)* and *h*_*slab*_*(T)*, it is possible to evaluate the wavelength shifts for each probe as function of temperature. To this aim, the LOF probe was numerically modeled with the finite element based commercial software Comsol Multyphisics (RF module), by following the approach described in ref.^22^. Coherently with our previous observations^22,38^, we considered the patterned hole sidewalls tilted of an angle δ = 40° with respect to fiber tip plane. The lattice period *a* and hole radius *r* were set to 680 nm and 190 nm respectively. Thickness of gold (*h*_*g*_) and chromium (*h*_*c*_) layers were set to 33 nm and 3 nm respectively. Gold and chromium RI were taken from ref.^49^ while optical fiber silica RI was modeled according to ref.^50^. The external medium is water (n = 1.33). MGs film was modeled with a uniform dielectric layer conformally deposited on the metallic grating with thickness *h*_*slab*_ and RI *n*_*slab*_. The achieved results (i.e. the wavelength shifts) are shown in Fig. 8a, and they are in reasonable agreement with the experimental ones (Fig. 5b).

The differences between results of Figs 5b and 8a, can be mainly related to the assumption that the volume of the MG particle dissolved in solution is the same of that of surface-attached particle. The fact that MGs are deposited (and thus constrained) on a surface, however, can slightly alter their swelling mechanism, thus affecting their volume variations as a function of the temperature. Moreover, the proposed model is essentially based on the assumption that the MGs deposition on a substrate form an equivalent (conformally deposited) slab, whose RI is given by an average between the RIs of the constituting materials. Obviously, this assumption is more valid when MGs are well packed among them over the surface as in the case of Probe2 and Probe3. On the contrary, when MGs are quite sparse on the surface (i.e. when they are deposited at low MGs concentrations) as in the case of Probe1, our model was not able to perfectly match experimental observations (i.e. the negative blue shift induced by the temperature increase). In this regard, it is important to remark that in our calculations we did not take into account the refractive index variations of both polymer (NIPAM) and liquid solution in which the probe are immersed. Both the refractive indexes are expected to decrease when the temperature increases^42,43^. Although it is not trivial, taking into account the thermo-optic effect (i.e. the refractive index changes induced by temperature) in our model is expected to improve the agreement between numerical and experimental results.

### Model implementation for responsivity enhancement

Once the model has been validated, it was also implemented in the same case study, to provide some design rules aimed at enhancing the probe responsivity. First, we plot in Fig. 8b, for each probe, the variation of *n*_*slab*_ as function of *h*_*slab*_ calculated according to eqs (7) and (8). Indeed, in the case of a MGs slab, RI and thickness change simultaneously, resulting in a RI pathline monotonically decreasing over the thickness. Clearly, the decreasing trend depends on the MGs film properties.

In Fig. 8b the RI pathlines are superimposed on a contour plot showing the resonant wavelength of the MGs-assisted LOF probe calculated for *all* possible combinations of *h*_*slab*_ and *n*_*slab*_. According to the contour plot, two different regimes of operation can be observed; for MGs thicknesses smaller than the decay length of the EM field at resonance, that is for *h*_*slab*_ < ~150 nm, the wavelength curves are well described by the classical plasmonic behavior. In fact, the increasing of both RI and thickness causes the plasmonic resonance wavelength red-shift^41^. For higher values of *h*_*slab*_ the resonance behavior is influenced by the occurrence of a Fabry-Perot like effect that induces a modulation of the plasmonic wavelength. Obviously, that effect is more evident for higher values of *n*_*slab*_.

With reference to Fig. 8b, it is interesting to note that the maximum sensitivities (in terms of wavelength shifts *vs* external stimulus) are achieved when the RI pathlines are *perpendicular* to the resonant wavelength curves. In the pure plasmonic regime, however, the MGs slab RI pathlines show a monotonically decreasing trend, which resembles that of the resonant wavelength curves. This result essentially limits the maximum sensitivity that it is possible to achieve with the MGs-assisted LOF probes analyzed in this work. As a limiting case, the MGs slab RI pathline could be in principle perfectly superimposed on one of the wavelength curves, thus annulling the sensitivity. It results clear that, for maximizing the plasmonic wavelength shift, both MGs layer and the metallic nanostructure underneath have to be designed in such a way to avoid this situation.

In this particular case study, our results suggest that, for increasing the sensitivity, it is convenient to use thicker MGs slab, for working *outside* of the pure plasmonic regime. The reason is that the damping effect due to the thickness sensitivity (that has an opposite sign with respect to the RI one) vanishes when MGs thickness reaches the values of about 150 nm. Achieving a larger MGs slab thickness can be accomplished by (i) further increasing the MGs density or (ii) by modifying the synthesis procedure (see the methods) in order to create a set of MGs with a larger hydrodynamic radius. It is important to remark that, for concentration larger than 0.5%, the resulting MGs film may not resemble a monolayer^22^. In that case, the swelling associated to a MGs multilayer structure is the result of complex interaction phenomena involving adjacent MGs that have to be taken into account in order to correctly evaluate the overall sensitivity. In any case, since the resonant wavelength curves become parallel for *h*_*slab*_ > 150 nm, further increasing the MGs slab thickness does not induce any sensitivity enhancement.

## Conclusions

In this work, we have proposed a method for modelling the optical properties of a MGs layer deposited on a substrate where resonant modes are excited. We have demonstrated that MGs layer essentially acts as a uniform equivalent dielectric slab characterized by RI and thickness whose values are strictly related among them, and vary as a function of the external stimuli applied. By keeping the general assumption that MGs particle volume is preserved after the deposition, we have derived simple equations that put in relation the geometrical and physical parameters of the MGs layer with respect to data of morphological analysis such as DLS characterizations (in liquid) and AFM measurements (in air). Specifically, these relationships are based on the definition of physical (i.e. the *polymer fraction parameter α*, inherently related to the MGs nature) and geometrical (such as the *thickness growth parameter β* and the *coverage factor γ*, related to MGs morphological characteristics when they are deposited on a surface) parameters. Our model has been validated in a particular case of study, where temperature-responsive MGs are deposited on a resonant plasmonic nanostructure integrated on top of the standard single mode optical fiber. The interaction of light with the MGs layer causes a resonant wavelength shift according to the swelling/shrinking behavior induced by the temperature change. The entity of the wavelength shift depends on the MGs density, which is strictly related to the MGs concentration in solution used during the fabrication procedure. We have demonstrated that, despite its simplicity, the model is accurate and reliable, providing a useful numerical tool for designing advanced photonic devices based on MGs integration. The model becomes less accurate when the MGs distribution is rather sparse. However, also in that case, the model provides a useful qualitative description of the light-MGs interaction phenomena. Although here applied for designing plasmonic LOF probes, the proposed method can be easily extended to other configurations where MGs are integrated onto flat or patterned (metallic or dielectric) surfaces. In this framework, current studies are devoted to apply the model to more complex optical structures, such as MGs-assisted plasmonic cavities that allow to effectively overcome the intrinsic RI/thickness opposite sensitivity trade-off^23^.

## Methods

### Materials

N-isopropylacrylamide, N,N′-methylenebisacrylamide (BIS; 99%), acrylic acid (AAc; 99%), ammonium persulfate (APS; 98%), and 3-amino phenyl boronic acid (APBA) hydrochloride (98%), Sodium dodecyl sulfate (SDS), sodium bicarbonate (NaHCO_3_) and sodium carbonate (Na_2_CO_3_) were purchased from Sigma Aldrich and used without further purification. 1-Ethyl-3-(3-dimethylaminopropyl) carbodiimide hydrochoride (EDC) and BupH 2-(Nmorpholino) ethanesulfonic acid (MES) buffered saline were purchased from Pierce through Thermo Scientific and used without further purification. All deionized water was filtered in order to reach a resistivity of 18.2MΩ cm and was obtained from a Milli-Q Plus system from Millipore.

### LOF probe fabrication

A 35 nm thick Au film has been deposited by means of electron beam evaporation on the cleaved end of a standard single mode optical fiber (Corning SMF-28); a thin (~2 nm) chromium film has been used for improving adhesion. During deposition, optical fibers have been mounted on a suitable holder, angled with respect to the evaporation direction, in such a way to promote Au deposition also along the fiber axis. This allows to avoid charging effect caused by the ion beam milling process. Finally, the square lattice of holes has been patterned on gold film by using the FEI Quanta 200 3D system. Beam currents and accelerating voltages have been set to 30 pA and 30 kV respectively.

### MGs synthesis

A solution of NIPAm (11.1 mmol), BIS (0.652 mmol) and SDS (0.035 g) in 99 mL deionized water was introduced in a flask equipped with a reflux condenser, gas inlet, and temperature controller. After heating the solution to 70 °C over ~1 hour, AAc (1. 3 mmol) monomer was then injected under nitrogen atmosphere. The polymerization was then initiated by adding a solution of APS (0.3 mmol) in 1 mL of deionized water under nitrogen and the reaction proceeded at 70 °C for 4 hours. The MGs solution was allowed to cool overnight and purified by using a dialysis tubes (12–14k nominal MWCO) in a deionized water solution. The purified (pNIPAM-co-AAc) MGs were collected and lyofilized.

### MGs functionalization

MGs were functionalized with boronic groups using APBA under EDC catalysis in pH 4,7 MES buffer. In details, the phenylboronic acid-functionalized (pNIPAM-co-APBA) MGs were obtained following the protocols described in ref.^19^. A solution of 250 mM of EDC and 125 mM APBA in pH 4.7 MES buffer was added to an aliquot of (pNIPAM-co-AAc) MGs (5 mL) cooled at 4 °C. The coupling reaction proceeded 12 hours at 4 °C. The functionalized MGs were cleaned by using a dialysis tubes (12–14k nominal MWCO) in a deionized water solution. Negative control samples were produced by reacting (pNIPAM-co-AAc) MGs with APBA only (without EDC) in pH 4.7 MES buffer.

### DLS measurements

Measurements were performed using a Dynamic Light Scattering system (Malvern Zetasizer Nano ZS instrument, 633 nm laser, 173° scattering angle) equipped with a temperature controller. To evaluate the thermo-responsivity behavior, for each temperature an equilibration time of 1200 s was fixed and a total of 5 measurements were conducted.

### Integration of MGs Film on the LOF probe

To prepare probe with different MGs concentration, the optical fiber tip was dipped for 1 h into MGs solution (200 µL) set at different concentrations (5%, 0.5%, 0.05%). The MGs layer was dried into an oven at 37 °C for 1 hour, placed in a deionized water bath at 30 °C overnight to eliminate non-specifically adsorbed MGs and then dried in air. The probes with MGs layer were equilibrated into a buffer solution (pH 4.7 MES) for 1 h at 4 °C and EDC and APBA were added to the buffer to reach a final concentration in solution of 250 mM and 125 mM, respectively. The reaction proceeded at 4 °C for 12 hours. Finally, all samples were cleaned in deionized water for 48 hour to pull-out any unreacted reagents.

### Optical characterization

Reflection spectra were measured by using the same setup described in ref.^22^. Measurements have been performed by illuminating the fiber probes with a broadband optical source (NKT SuperK COMPACT) and redirecting the reflected light (*via* a 2 × 2 directional coupler) to an optical spectrum analyzer. The reflectance spectrum has been normalized by that of the source, obtained by measuring the light transmitted through the coupler using another optical spectrum analyzer.

## Electronic supplementary material

Supplementary Information - Light-microgel interaction in resonant nanostructures
