# Supplementary material for: Light-microgel interaction in resonant nanostructures
Source: Sci Rep. 2018 Jun 19;8:9331. doi: 10.1038/s41598-018-27197-4 (PMC6008304; doi:10.1038/s41598-018-27197-4)
Supplement: Supplementary file 1 — Supplementary Information - Light-microgel interaction in resonant nanostructures [file 41598_2018_27197_MOESM1_ESM.pdf]

Supplementary Information

Light-microgel interaction in resonant nanostructures

M. Giaquinto[1], A. Ricciardi[1], A. Aliberti[1], A. Micco[1], E. Bobeico[2], M. Ruvo[3], A. Cusano[1,]*

[1] Optoelectronics Group, Department of Engineering, University of Sannio, I-82100, Benevento, Italy

[2] ENEA, Portici Research Center, P.le E. Fermi 1, I-80055 Portici, Napoli, Italy

[3] Institute of Biostructure and Bioimaging, National Research Council, I-80143, Napoli, Italy

[*]*corresponding author: a.cusano@unisannio.it*

S1. Definition of the *single* microgel particle refractive index

The single MG refractive index is expressed as:

$$n_{MGs}=\frac{n_pV_p+n_sV_s}{V}=\frac{n_p\alpha V_{\min}+n_s\left(V-\alpha V_{\min}\right)}{V}=\alpha\cdot(n_p-n_s)\cdot\frac{V_{\min}}{V^3}+n_s=\alpha\cdot(n_p-n_s)\cdot\frac{\frac{4}{3}\pi R_{\min}^3}{\frac{4}{3}\pi R^3}+n_s=$$

$$=\alpha\cdot(n_p-n_s)\cdot\frac{R_{\min}^3}{R^3}+n_s$$

where we assume:

$V=V_p+V_s=\frac{4}{3}\pi R^3$: MG volume in swollen state

$V_p=\alpha V_{\min}$: Volume of the polymeric component inside the single MG

$V_s=V-\alpha V_{\min}$: Volume of the liquid component inside the single MG

$V_{\min}=\frac{4}{3}\pi R_{\min}^3$: minimum volume assumed by a single MG swollen in solution

S2. Definition of the microgel *slab* refractive index

The MG slab is defined as:

$$n_{slab}=\gamma\cdot n_{MGs}+(1-\gamma)\cdot n_s=\gamma\cdot\alpha\cdot(n_p-n_s)\cdot\frac{R_{\min}{}^3}{R^3}+\gamma\cdot n_s+n_s-\gamma\cdot n_s=\gamma\cdot\alpha\cdot(n_p-n_s)\cdot\frac{R_{\min}{}^3}{R^3}+n_s$$

S3. Evaluation of the parameter α

The parameter α is calculated as follows:

$$\alpha = \frac{V_p}{V_{\min}} = \frac{\frac{h}{6}\pi\left(3r_b^2 + h^2\right)}{\frac{4}{3}\pi R_{\min}^3} = \frac{h^3 + 3hr_b^2}{8R_{\min}^3}$$

Note that V_p is evaluated by assuming that the deposited MG in air has the shape of a spherical cap, with base radius r_b, and height h.

S4. Evaluation of the parameter γ

The factor $\overline{\gamma}$ is defined as:

$$\overline{\gamma} = \frac{A_{tot}}{A_{slab}} = \frac{N_{MGs}A}{A_{slab}} = \frac{N_{MGs}\pi\overline{r}^2}{A_{slab}}$$

note that A is the area occupied by the single MG at half height.

By inverting the above equation, we find:

$$\frac{N_{MGs}}{A_{slab}} = \frac{\overline{\gamma}}{\pi\overline{r}^2}$$

Once defined $\frac{N_{MGs}}{A_{slab}}$, γ is calculated as

$$\gamma = \frac{V_{tot}}{V_{slab}} = \frac{N_{MGs}V}{A_{slab}h_{slab}} = \frac{\overline{\gamma}}{\pi\overline{r}^2}\frac{\frac{4}{3}\pi R^3}{\beta \cdot R} = \frac{4}{3}\left(\frac{R}{\overline{r}}\right)^2\frac{\overline{\gamma}}{\beta}$$

S5. Evaluation of the parameter *β*

When γ<1, the single deposited MG can be modeled as a spherical cup with volume V_{dep}.

Our model is based on the assumption that the single MG volume does not change when it is deposited on a surface, that means:

$$V_{dep} = V \Leftrightarrow \frac{h_{slab}}{6}\pi\left(3r_b^2 + h_{slab}^2\right) = \frac{4}{3}\pi R^3 \Leftrightarrow \overbrace{h_{slab}^3 + 3r_b^2 h_{slab} - 8R^3 = 0}^{*}$$

The (unique) *real* solution of the equation (*) is:

$$h_{slab} = \left(4R^3 + \sqrt{r_b^6 + 16R^6}\right)^{1/3} - \frac{r_b^2}{\left(4R^3 + \sqrt{r_b^6 + 16R^6}\right)^{1/3}}$$

The first order of the Taylor series of the above equation evaluated around r_b is given by:

$$h_{slab,1^{st}} = 2.4R - 0.92r_b$$

and thus β can be eventually approximated as

$$\beta \approx 2.4 - 0.92\frac{r_b}{R}, \quad \gamma < 1$$

Note that this approximation is valid over a large range of R as shown in **Figure S1,** where both the rigorous and the approximated solutions of h_{slab} are shown as a function of R (for three arbitrary values of r_b, i.e. 50nm, 70nm and 90nm).

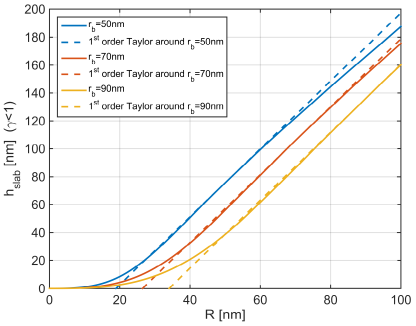

Figure S1: Comparison between rigorous and approximated solution of h_{slab} in the case of γ<1.

As described in the main text, when γ=1, β is:

$$\beta = \bar{\gamma}\frac{4}{3}\left(\frac{R}{r}\right)^2, \ \gamma = 1.$$
